# Chemical constituents of industrial hemp roots and their anti-inflammatory activities

**DOI:** 10.1186/s42238-022-00168-3

**Published:** 2023-01-16

**Authors:** Shijie Huang, Huifang Li, Jun Xu, Huihao Zhou, Navindra P. Seeram, Hang Ma, Qiong Gu

**Affiliations:** 1 Research Center for Drug Discovery, School of Pharmaceutical Sciences, Sun Yat-sen University, 510006 Guangzhou, China; 2 Bioactive Botanical Research Laboratory, Department of Biomedical and Pharmaceutical Sciences, College of Pharmacy, University of Rhode Island, 02881 Kingston, RI USA

**Keywords:** Hemp, *Cannabis sp*. phytochemicals, Cannabinoids, Inflammasome

## Abstract

**Objective:**

Although the chemical constituents of the aerial parts of *Cannabis* have been extensively studied, phytochemicals of *Cannabis* roots are not well characterized. Herein, we investigated the chemical constituents of industrial hemp (*Cannabis sativa* L.) roots and evaluated the anti-inflammatory activities of phytochemicals isolated from the hemp roots extract.

**Methods:**

An ethyl acetate extract of hemp roots was subjected to a combination of chromatographic columns to isolate phytochemicals. The chemical structures of the isolates were elucidated based on spectroscopic analyses (by nuclear magnetic resonance and mass spectrometry). The anti-inflammatory effects of phytochemicals from hemp roots were evaluated in an anti-inflammasome assay using human monocyte THP-1 cells.

**Results:**

Phytochemical investigation of hemp roots extract led to the identification of 32 structurally diverse compounds including six cannabinoids (1–6), three phytosterols (26–28), four triterpenoids (22–25), five lignans (17–21), and 10 hydroxyl contained compounds (7–16), three fatty acids (29–31), and an unsaturated chain hydrocarbon (32). Compounds 14–21, 23, 27, and 32 were identified from the *Cannabis* species for the first time. Cannabinoids (1–5) reduced the level of cytokine tumor necrosis-alpha (by 38.2, 58.4, 47.7, 52.2, and 56.1%, respectively) and 2 and 5 also decreased the interleukin-1β production (by 42.2 and 92.4%, respectively) in a cell-based inflammasome model. In addition, non-cannabinoids including 11, 13, 20, 25, 29, and 32 also showed selective inhibition of interleukin-1β production (by 23.7, 22.5, 25.6, 78.0, 24.1, 46.6, and 25.4%, respectively) in THP-1 cells.

**Conclusion:**

The phytochemical constituent of a hemp roots extract was characterized and compounds from hemp roots exerted promising anti-inflammatory effects.

**Supplementary Information:**

The online version contains supplementary material available at 10.1186/s42238-022-00168-3.

## Introduction

The medicinal uses of *Cannabi*s roots for a variety of maladies are supported by empirical practice and emerging scientific evidence. The ancient Chinese herbology Compendium of Materia Medica (“Bencao Gangmu” in Chinese or Great Pharmacopoeia) was one of the earliest books that documented the use of hemp (*Cannabis sativa* L.) roots as a folk medicine to soothe the pain. The traditional Chinese medicine system also uses hemp roots as a common practice to treat Reqi (“hot air” or heatiness)-related symptoms (e.g., mouth ulcers) by indigenous people from Southwest China regions. Modern scientific studies provide more rigorous evidence of the biological activities of cannabis roots. Several pre-clinical studies reported that cannabis root extracts exert various pharmacological effects including anti-inflammatory, estrogenic, liver protective, and anti-cancer activities (Elhendawy et al., 2019; Kornpointner et al. 2021; Lima, et al., 2021; Ryz et al., 2017). Notably, there are fewer reported studies on cannabis roots as compared to the plant’s aerial parts including the inflorescences and leaves. This is possibly due to the lower density of glandular trichomes in cannabis roots, which leads to a lower level of cannabinoids biosynthesis (Stout et al., 2012). Moreover, the mechanisms of actions of enzymes that contribute to the biosynthesis of phytochemicals in the roots of cannabis are not fully elucidated (Sirikantaramas and Taura 2017). Nevertheless, several studies on hemp roots have reported the isolation and identification of several phytochemicals including cannabinoid-type of compounds, such as cannabidiol (CBD), tetrahydrocannabinol (THC), cannabichromene, cannabigerol, and cannabigerolic acid, as well as non-cannabinoid-type of compounds including flavonoids, triterpenes, polyphenols, and alkaloids (Stout et al., 2012). In addition, a study characterized the cannabinoids content (ranging between 0.001 and 0.004%) in three chemovars, which was lower than the other chemotypes of compounds such as sterols (0.06–0.09%) and triterpenoids (0.13–0.24%) (Jin et al. 2020). Notably, some unique compounds have been isolated from cannabis roots. For instance, a phenolic amide, namely, N-trans-coumaroyltyramine, was isolated from the roots of a *Cannabis sp.* grown in Mississippi, USA (Pollastro et al., 2018). In addition, several arylnapthalene-type of compounds including cannabisins B and G were only found in fruits and roots of cannabis (Sakakibara et al., 1995). However, to date, the phytochemical constituents of hemp roots and their biological activities are not been fully investigated. Our group has initiated a program to systematically investigate the biological activities of non-psychedelic cannabinoids (Liu et al., 2021, 2020, 2022; Ma, Li, et al., 2021; Ma, Xu, et al., 2021; Puopolo et al., 2022). During this course, we revealed that the anti-inflammatory activity of CBD is associated with its inhibitory effect on the activation of NLR-family-pyrin-domain-containing 3 (NLR3) inflammasome, which is a vital molecular target for the production of pro-inflammatory cytokine (i.e., interleukin 1 beta; IL-1β) (Liu et al. 2020). However, the phytochemical constituents of hemp roots from the region of Southwest China (i.e., Yunnan Province) and the inhibitory effects of cannabinoids from hemp roots are still not clear. Herein, we report the isolation and identification of phytochemicals of hemp roots as well as the biological evaluation of isolated phytochemicals from hemp roots using a cell-based inflammasome model.

## Materials and methods

### Chemical reagents

The roots of industrial hemp were provided by Mawang Shenzhen Co. Ltd. (Shenzhen, China) and a voucher sample (No. MG20191230) is deposited in the School of Pharmaceutical Sciences of Sun Yat-Sen University. Lipopolysaccharides (LPS), nigericin, phorbol 12-myristate 13-acetate (PMA), and phosphate-buffered saline were purchased from Sigma-Aldridge Chemical Co (St. Louis, MO, USA).

### Extraction and isolation

Dried hemp roots (20 kg) were pulverized and extracted with 95% EtOH (3 × 150 L) for 48 h at room temperature. The solvent was evaporated in vacuo, and the dried ethyl acetate extract (732 g) was suspended in water and partitioned with ethyl acetate 3 times to obtain an ethyl acetate extract (350 g). This extract was chromatographed over a combination of columns (silica gel, Sephadex LH-20, thin-layer chromatography, and semi-preparative HPLC) using various eluting solvents to obtain following purified compounds (see detailed separation and purification in the Supplementary Data): compounds 1 (4 mg, 0.00002%), 2 (31 mg, 0.000155%), 3 (12 mg, 0.00006%), 4 (7 mg, 0.000035%), 5 (9 mg, 0.000045%), 6 (6 mg, 0.00003%), 7 (12 mg, 0.00006%), 8 (90 mg, 0.00045%), 9 (48 mg, 0.00024%), 10 (22 mg, 0.00011%), 11 (125 mg, 0.000625%), 12 (8 mg, 0.00004%), 13 (9 mg, 0.000045%), 14 (11 mg, 0.000055%) 15 (30 mg, 0.00015%), 16 (6 mg, 0.00003%), 17 (43 mg, 0.000215%), 18 (89 mg, 0.000445%), 19 (8 mg, 0.00004%), 20 (67 mg, 0.000335%), 21 (314 mg, 0.00157%), 22 (28 mg, 0.00014%), 23 (17 mg, 0.000085%), 24 (239 mg, 0.001195%), 25 (1.3 g, 0.0065%), 26 (15 mg, 0.000075%), 27 (14 mg, 0.00007%), 28 (18 mg, 0.00009%), 29 (31 mg, 0.000155%), 30 (51 mg, 0.000255%), 31 (25 mg, 0.000125%), and 32 (14 mg, 0.00007%).

### Cell culture

Human monocyte THP-1 cells were purchased from the American Type Culture Collection (ATCC, Rockville, MD, USA) and cultured in the Roswell Park Memorial Institute 1640 medium supplemented with 10% fetal bovine serum (Gibco, Life Technologies, Gaithersburg, MD, USA).

### Inflammasome inhibition assay

The anti-inflammasome effects of cannabinoids isolated from hemp root were evaluated by measuring the production of lipopolysaccharide (LPS)- and nigericin-induced secretion of pro-inflammatory cytokines including interleukin-1β (IL-1β) and tumor necrosis factor-α (TNF-α) using a previously reported method (Liu et al. 2020). Briefly, THP-1 cells were seeded at a density of 5 × 10^4^ cells per well in a 48-well plate and differentiated with phorbol 12-myristate 13-acetate (PMA) (25 nM) for 48 h. Then, culture medium was removed and replaced with PMA-free medium for 24 h. Next, LPS (100 ng/mL) was added and incubated for 4 h, followed by adding hemp root samples (compounds 1–5; 50 µM) and further incubated for 1 h. Then, nigericin (10 µM) was added and incubated with the cells for 4 h. The cell culture supernatant was collected to measure the levels of IL-1β and TNF-α using the ELISA kits (BioLegend, San Diego, CA, USA).

### Statistical analysis

Data are presented as mean ± standard deviation of values from three replicated experiments. Statistical analysis was performed using the GraphPad Prism9 software (GraphPad, La Jolla, CA, USA) with a one-way analysis of variance with multiple comparisons. The significance was noted as *p* < 0.05 (*), *p* < 0.01 (**), *p* < 0.001 (***), and *p* < 0.0001 (****).

## Results and discussion

### Isolation and identification of phytochemicals isolated from hemp roots

**Fig. 1:**
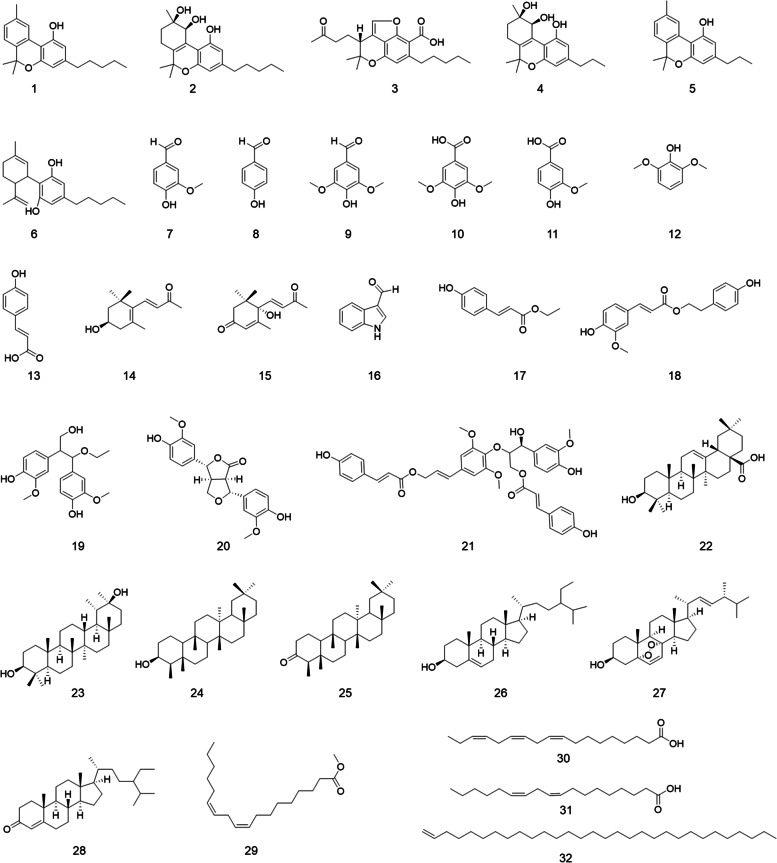
Chemical structures of compounds 1–32 including cannabinoids (1–6) and non-cannabinoids (7–32) isolated from hemp roots

Thirty-two phytochemicals (1–32) were isolated from the hemp roots extract, and their chemical structures (Fig. 1) were elucidated on the basis of spectroscopic analysis (by NMR and MS) and comparisons of the literature reported values as follows: cannabinol (1) (Wang et al., 2017); (9*R*,10* S*)-6,6,9-trimethyl-3-pentyl-7,8,9,10-tetrahydro-6* H*-benzo[*c*]chromene-1,9,10-triol (2) (Li et al., 2020); (-)-7*R*-cannabicoumarononic acid A (3) (Radwan et al., 2009); (9*R*,10* S*)-6,6,9-trimethyl-3-propyl-7,8,9,10-tetrahydro-6* H*-benzo[*c*]chromene-1,9,10-triol (4) (Li et al., 2020); cannabivarin (5) (Wang et al., 2017); cannabidiol (6) (Marchetti et al. 2019); 4-hydroxy-3-methoxybenzaldehyde (7) (Ito et al., 2001); 4-hydroxybenzaldehyde (8) (Bouaicha et al., 1994); 4-hydroxy-3,5-dimethoxybenzaldehyde (9) (Xue et al., 2013); syringic acid (10) (Queirós et al., 2020); vanillic acid (11) (Xu et al. 2022); 2,6-dimethoxyphenol (12) (Goda et al., 1987); (2*E*)-3-(4-hydroxyphenyl)-2-propenoic acid (13) (Seal, 2013); (3* S*)-3-hydroxy-*β*-ionone (14) (DellaGreca et al., 2004); (-)-dihydrovomifoliol (15) (Bennett et al., 1990); 1* H*-indole-3-carboxaldehyde (16) (Wang et al., 2012); 3-(4-hydroxyphenyl)-2-propenoic acid ethyl ester (17) (Pizzolatti et al., 2006); 4-hydroxyphenethyl trans-ferulate (18) (Fujita et al., 1995); 1,2-diguaiacyl-1-ethoxy-3-propanol (19) (Mohammed et al., 2022); (+)-4-ketopinoresinol (20) (Chen et al., 2012); dadahol A (21) (Devanathan and Stalin 2020); (+)-oleanolic acid (22) (He et al., 2021), epipseudotaraxastanediol (23) (Hinge et al., 1966); 3-epifriedelinol (24) (Sousa et al., 2012); 9,12-octadecadienoic acid (9*Z*,​12*Z*)​-​friedelin (25) (Zhou & Guo, 2009); *β*-sitosterol (26) (Kadowaki et al., 2003); 5*α*,8*α*-epidioxy-(22*E*,24*R*)-ergosta-6,22-dien-3*β*-ol (27) (Gao et al., 2007); stigmast-​4-​en-3-one (28) (Smania et al., 2003); 9,​12-​octadecadienoic acid (9*Z*,​12*Z*)​-​methyl ester (29) (Huh et al., 2010); *α*-linolenic acid (30) (Kishino et al., 2003); linoleic acid (31) (Yoon et al., 2015); and dotriacont-1-ene (32) (Chen et al., 2010). The isolated phytochemicals can be divided into two classes, the cannabinoid-type (1–6) and the non-cannabinoid-type (7–32). Among the cannabinoids, compound 1 (cannabinol) is a derivative of tetrahydrocannabinol, which is the major psychedelic (hallucinogenic substance triggering non-ordinary states of consciousness, also referred to as psychoactive or psychotropic) cannabinoid found in cannabis (Sampson, 2021). In contrast to tetrahydrocannabinol, cannabinol only shows mild psychedelic effects (Rhee et al., 1997). Other cannabinoid-type compounds isolated from the hemp roots were non-psychedelic (Fig. 1).

Apart from cannabinoid-type compounds (1–6), several non-cannabinoid compounds were identified from hemp roots. These phytochemicals included three phytosterols (26–28), four triterpenoids (22–25), and five lignans (17–21). Several compounds were isolated and identified from *Cannabis sp*. for the first time as follows. Compounds 20, 21, 23, and 27 were reported from the *Cannabis* species for the first time. Additionally, 10 hydroxyl-contained compounds (7–16) were isolated from the hemp roots. Among these, compounds 14–19 were identified from *Cannabis* for the first time. Moreover, three fatty acids (29–31) and an unsaturated hydrocarbon (32) were identified in *Cannabis* for the first time.

### Anti-inflammatory effects of phytochemicals isolated from hemp roots

**Fig. 2:**
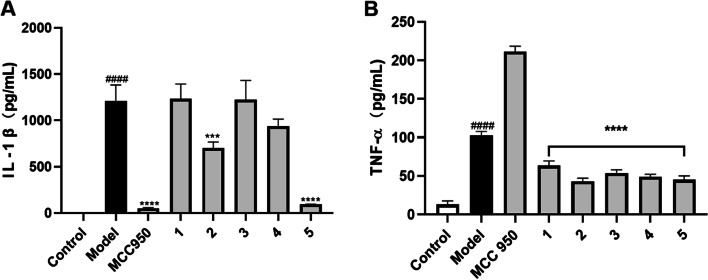
Anti-inflammatory effects of cannabinoids (1–5) isolated from hemp roots. Inflammasome activation in THP-cells was induced by the stimulation with LPS-nigericin. Effects of 1–5 (at 50 µM) on the production of pro-inflammatory cytokines including IL-1β (**A**) and TNF-α (**B**). Data are presented as means ± standard deviation from samples with three replicated experiments (*n* = 3). Statistical analysis was performed with a one-way analysis of variance with multiple comparisons and the significance was noted as: ***p < 0.001, ****p < 0.0001 vs. the model (LPS-nigericin stimulated) group and ####*p* < 0.0001 vs. the control group

Our group has previously reported that CBD, the major non-psychedelic cannabinoid in cannabis, confers the anti-inflammatory effect via a specific mechanism, namely, inhibition of inflammasome activation (Liu et al. 2020). However, it is not clear whether other cannabinoids from the hemp roots are inflammasome inhibitors. Thus, an inflammasome inhibition assay was performed to evaluate the anti-inflammatory effects of cannabinoids isolated from hemp roots. The production of IL-1β and TNF-α in inflammasome inducers (LPS and nigericin) stimulated THP-1 cells were significantly increased as compared to the control group (6.1 vs. 1211.6 and 13.0 vs. 102.6 pg/mL, respectively), suggesting that inflammasome in THP-1 cells was activated by the stimulation with LPS and nigericin. Compounds 2 and 5 (50 µM) counteracted LPS-nigericin-induced production of IL-1β by 42.2% and 92.4%, respectively (Fig. 2A). Cannabinoids (1–5) reduced the level of TNF-α by 38.2%, 58.4%, 47.7%, 52.2%, and 56.1%, respectively (Fig. 2B). To confirm whether the anti-inflammatory of these cannabinoids were selective inhibitors of the inflammasome, we used a small molecule with known a selective inflammasome inhibitor effect (Wu et al., 2020), namely, MCC950, as a positive control. MCC950 (10 µM) showed a specific inhibitory effect against the activation of the inflammasome by reducing the level of IL-1β (by 95.8%) without decreasing the level of TNF-α. Additionally, a small molecule with inhibitory effects on the production of TNF-α (DaSilva et al., 2019; Xu et al., 2018), namely, urolithin A, was included as a positive control (as a TNF-α inhibitor) to validate the anti-inflammatory assay. Urolithin A reduced the level of TNF-α by 51.7% as compared to the model group. With these validated bioassays, we were able to evaluate the selective anti-inflammasome activity of the isolates. Although our previously reported study showed that CBD is a specific inflammasome inhibitor (Liu et al. 2020), cannabinoids isolated from hemp roots extract exerted general anti-inflammatory effects rather than specific inhibition of inflammasome activation. The inhibitory effects of cannabinoids from hemp roots on the secretion of pro-inflammatory cytokine (TNF-α) may be accounted for the overall anti-inflammatory activities of *Cannabis* roots extracts in previously reported studies (Lima, et al., 2021; Ryz et al., 2017). However, several factors that may influence the anti-inflammatory effects of tested cannabinoids should be examined. For instance, the cytotoxicity of these cannabinoids in THP-1 cells may be accounted for the reduction of the pro-inflammatory cytokines. Thus, we assessed the viability of THP-1 cells treated with cannabinoids. Compounds 1–4 showed no significant cytotoxic effect (viability > 95%) at the concentration used for the anti-inflammasome assay (50 µM) whilst cannabivarin (5) reduced the cell viability by 54.7%, which possibly contributed to its suppressive effect on the production of IL-1β and TNF-α (see cell viability data in the Supplementary Data Figure S1). Therefore, further mechanistic investigation on the anti-inflammatory effects of cannabinoids from hemp roots should be conducted at non-cytotoxic concentrations. Nevertheless, this is the first study on the specific anti-inflammasome activity of cannabinoids isolated from hemp roots.

**Fig. 3:**
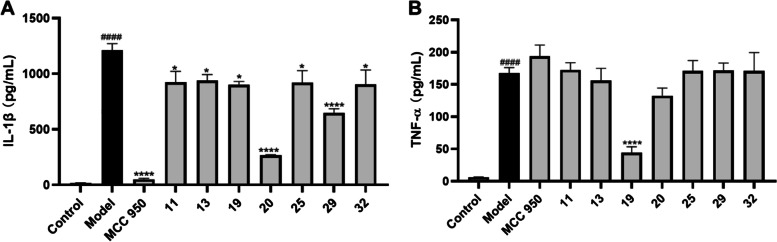
Anti-inflammatory effects of non-cannabinoids isolated from hemp roots (11, 13, 19, 20, 25, 29, and 32). Inflammasome activation in THP-cells was induced by the stimulation with LPS-nigericin. Effects of 11, 13, 19, 20, 25, 29, and 32 (at 50 µM) on the production of pro-inflammatory cytokines including IL-1β (**A**) and TNF-α (**B**). Data are presented as means ± standard deviation from samples with three replicated experiments (*n* = 3). Statistical analysis was performed with a one-way analysis of variance with multiple comparisons and the significance was noted as: *p < 0.005, ****p < 0.0001 vs. the model (LPS-nigericin stimulated) group and ####*p* < 0.0001 vs. the control group

As none of the cannabinoids showed a selective inhibitory effect on inflammasome, we expanded the test samples for the anti-inflammation assay and further evaluated the suppressive effects of other isolates (7–32) on the production of IL-1β (Figure S2). At a concentration of 50 µM, compounds 11, 13, 19, 20, 25, 29, and 32 reduced the production of IL-1β by 23.7, 22.5, 25.6, 78.0, 24.1, 46.6, and 25.4%, respectively (Fig. 3A). These active compounds belong to different chemotypes including simple phenolics (11 and 13), lignans (19 and 20), triterpenoid (25), and unsaturated fatty acids (29 and 32). These active compounds were further assayed in the TNF-α inhibition assay, which showed that, apart from 19, they were inactive in reducing TNF-α production (Fig. 3B). Thus, compounds 11, 13, 20, 25, 29, and 32 showed an inhibitory effect on the activation of the inflammasome and they may contribute to the overall anti-inflammatory activity of cannabis (Lima, et al., 2021; Ryz et al., 2017). The selective inhibitory effects of compounds 11, 13, 20, 25, 29, and 32 on TNF-α production were in agreement with previously reported studies showing that these types of compounds were inflammasome inhibitors (Guo et al., 2017; L’homme et al., 2013; Ma, Huang, et al., 2021; Villar-Lorenzo et al., 2016). Further mechanistic studies are warranted to elucidate whether other pathways (e.g., via the antagonistic effect against the P2 × 7 receptor (Liu et al. 2020)) were involved in the anti-inflammasome effects of these compounds from *Cannabis* roots.

In summary, a phytochemical investigation of a hemp roots extract led to the identification of 32 compounds including six cannabinoids (1–6) and 26 non-cannabinoid compounds. Isolates 14–21, 23, 27, and 32 were reported from *Cannabis* for the first time. Cannabinoids from the hemp roots extract exerted anti-inflammatory effects by reducing pro-inflammatory cytokines in an inflammasome model with THP-1 cells. Cannabinoids 2 and 5 reduced LPS-nigericin-induced production of cytokines IL-1β (by 42.2% and 92.4%, respectively) and TNF-α (by 58.4% and 56.1%, respectively). In addition, non-cannabinoids type compounds including 11, 13, 19,20, 25, 29, and 32 also showed promising anti-inflammatory effects. Findings from the current study expanded the understanding of the phytochemical constituents of cannabis roots and their anti-inflammatory effects, which may provide useful information for the utilization of cannabis roots as bioactive natural products.

## Supplementary Information

**Additional file 1.**

## Data Availability

The data used for this study are available from the corresponding authors with a reasonable request.

## Abbreviations

CBD: Cannabidiol
HPLC: High-performance liquid chromatography
IL**-**1β: Interleukin 1 beta
LPS: Lipopolysaccharides
MS: Mass spectrometry
NLR3: NLR family pyrin domain containing 3
NMR: Nuclear magnetic resonance
PMA: Phorbol 12-myristate 13-acetate
THC: Tetrahydrocannabinol
TNF-α: Tumor necrosis factor-α
