# Supplementary material for: Chemical constituents of industrial hemp roots and their anti-inflammatory activities
Source: J Cannabis Res. 2023 Jan 16;5:1. doi: 10.1186/s42238-022-00168-3 (PMC9841654; doi:10.1186/s42238-022-00168-3)
Supplement: Supplementary file 1 — Additional file 1. [file 42238_2022_168_MOESM1_ESM.docx]

**Supplementary Data**

Chemical constituents of industrial hemp roots and their anti-inflammatory activities

Shijie Huang^1ǂ^, Huifang Li^2ǂ^, Jun Xu^1^, Huihao Zhou^1^, Navindra P. Seeram^2^,

Hang Ma^2^*, Qiong Gu^1^*

^1^Research Center for Drug Discovery, School of Pharmaceutical Sciences, Sun Yat-sen University, Guangzhou 510006, China

^2^Bioactive Botanical Research Laboratory, Department of Biomedical and Pharmaceutical Sciences, College of Pharmacy, University of Rhode Island, Kingston, Rhode Island 02881, USA

ǂ Equal contribution

*Corresponding authors

Tel.: +86 13450420126; E-mail address: guqiong@mail.sysu.edu.cn (Q.G.)

Tel.: +1 401 874 7654; E-mail address: hang_ma@uri.edu (H.M.)

**Contents**

S1. General experimental procedures

S2. Plant material

S3. Extraction and isolation

S4. Cell viability assay

Figure S1.

Figure S2.

**S1. General experimental procedures**

NMR spectra were recorded on Bruker AM-500 and Bruker AM-400. Chemical shifts (δ) were expressed in ppm with reference to the solvent signals. MPLC was performed on a Biotage Isolera one and semipreparative HPLC separations were conducted on a LC-20AT Shimadzu liquid chromatography system connected with an SPD-M20A diode array detector, with a Welch AQC18 column (5 μm, 250 × 10 mm). Silica gel (200–300 Mesh Marine Chemical Ltd, Qingdao, China), MCI gel (CHP20P, 75–150 μm, Mitsubishi Chemical Corporation, Tokyo, Japan), and Sephadex LH-20 (GE Healthcare Bio-Sciences AB, Sweden). All solvents used were of analytical grade (Guangzhou Chemical Reagents Company, Ltd.).

**S2. Plant material**

The roots of *C. sativa* L. (Industrial Hemp) were supplied by Mawang Cop. (Yunnan, China) in December 2019 and identified by Associate Professor Gu Qiong of the School of Pharmaceutical Sciences of Sun Yat-Sen University. The voucher sample (No. 20191230) has been stored in the School of Pharmaceutical Sciences of Sun Yat-Sen University.

**S3. Extraction and isolation**

The dried hemp roots (20 kg) were powdered and extracted with 95% EtOH (3 × 150 L) for 48 hours at room temperature for three times. The solvent was evaporated in vacuo, and the dried EtOH extract (732 g) was suspended in water and partitioned with EtOAc for three times to obtain EtOAc soluble residue (350 g). The EtOAc-soluble part (350 g) was chromatographed over silica gel column using petroleum ether/EtOAc (from 100:0 to 0:1, v/v) as eluents to obtain five fractions (Fr.A ‒ Fr.E). Fraction A (40 g) was subjected to silica gel column chromatography (CC) using a solvent system of petroleum ether−dichloromethane (from 100:0 to 0:1, v/v) as solvents to obtain seven subfractions (A1−A7). Fr. A2 and A4 was purified using NP-HPLC (1% isopropanol in n-hexane, 1.0 mL/min) to yield **32** (14 mg) and **6** (6 mg). Fraction A5 afforded compound **25** (1.3 g) by silica gel column chromatography (CC) using a solvent system of petroleum ether−dichloromethane (from 100:0 to 0:1, v/v). Fraction B (23 g) was loaded onto a silica gel CC using a solvent system of petroleum ether−dichloromethane (from 50:0 to 0:1, v/v) as solvents to obtain six subfractions (B1−B6). Fr. B2, B3, B4, and B5 was further purified using NP-HPLC (2 or 3% isopropanol in n-hexane, 1.0 mL/min) to yield compounds **5** (9 mg), **24** (239 mg), **28** (18 mg) and **29** (31 mg).

Fraction C (40 g) was subjected to an RP-18 column eluting with CH_3_OH−H_2_O (30 to 100%, v/v) to afford five fractions (C1−C5). Fr.C1 was further separated by silica gel CC with petroleum ether−EtOAc (from 40:1 to 1:1, v/v) as eluents to obtain five subfractions (C1a−C1e). Fr.C1b was purified using silica gel CC with petroleum ether/acetone (from 50:1 to 1:1, v/v) and thin-layer chromatography to yield compounds **7** (12 mg), **8** (90 mg), and **12** (8 mg). Fr. C1d afforded compound **17** (43 mg) by HPLC (75% CH_3_CN in H_2_O, 1.0 mL/min). Fr. C2 was separated by silica gel CC using a petroleum ether−EtOAc gradient (from 20:1 to 0:1, v/v) to give four subfractions (C2a−C2d). Fr.C2c and C2d was purified by semi-preparative HPLC using a mobile phase of 75% CH_3_CN in H_2_O (1.5 mL/min) to afford **1** (4 mg), **30** (51 mg), **31** (25 mg), **26** (15 mg) and **22** (28 mg). Fr.C3 was fractionated by silica gel CC to give five subfractions (C3a-C3e). Fr.C3b was subjected to a silica gel column eluting with a gradient of dichloromethane/CH_3_OH (from 100:0 to 10:1, v/v) to yield **23** (17 mg) and **27** (14 mg).

Fraction D (39 g) was loaded onto a reversed-phase C18 silica gel column eluting with MeOH−H_2_O (30−100%, v/v) to obtain five fractions (D1−D5). Fr. D1 was further separated by silica gel CC with petroleum ether−EtOAc (from 20:1 to 0:1, v/v) as eluents to obtain seven subfractions (D1a-D1g). Fr. D1a was subjected to a silica gel column eluting with a gradient of petroleum ether/EtOAc (from 20:1 to 5:1, v/v) to yield compounds **19** (8 mg) **14** (11 mg), and **15** (30 mg). Fr. D1b was purified using Sephadex LH-20 column with CH_3_OH as eluent to yield compound **10** (22 mg). Fr. D1c afforded compounds **20** (67 mg) and **9** (48 mg) by HPLC (50% CH_3_CN in H2O, 1.0 mL/min). Fr. D1d afforded compounds **16** (6 mg) and **11** (125 mg) by silica gel CC using petroleum ether−EtOAc (from 20:1 to 4:1, v/v). Fr. D1g was purified by thin-layer chromatography using petroleum ether/EtOAc (10:1) to obtain compound **13** (9 mg). Fr. D2 was separated by silica gel CC using a petroleum ether−acetone gradient (from 30:1 to 1:1, v/v) to give six subfractions (D2a-D2f). Fr. D2b was fractionated on a Sephadex LH-20 column with CH_3_OH as eluent to afford subfractions D2b-1 to D2b-3, which were purified by semi-preparative HPLC (60% CH_3_CN in H_2_O, 1.0 mL/min) to afford compounds **2** (31 mg), **3** (12 mg), and **4** (7 mg). Fr. D2c was subjected to a silica gel column eluting with a dichloromethane/CH_3_OH (from 100:1 to 1:1, v/v) to yield compound **21** (314 mg). Fr. D2d was fractionated by silica gel CC to give four subfractions (D2d-1−D2d-4). Fr. D2d-2 was separated by semi-preparative HPLC (50% CH_3_CN in H_2_O, 1 mL/min) to give compound **18** (89 mg).

**S4. Cell viability assay**

THP-1 cells were seeded in 96-well plates at 1.0×10^4^ cells per well at 37 ℃ with 5% CO_2_ and allowed to attach for 48 h with PMA (25 nM). Then medium containing PMA was replaced by a fresh medium without PMA for 24 h, followed by adding test samples (compounds **1-5**; 50 μM) with or without LPS (100 ng/mL) and further incubated for 24 h. Next, the MTT reagent in cell culture medium (110 µL) was added to each well and incubated for 4 h. Next, the medium was removed and DMSO (100 µL) was added to each well, for 10 min. The absorbance of each well was then recorded using a plate reader at 570 nm.

**Figure S1.** Cell viability of active compounds from the inflammatory assays (**1**-**5**, **11**, **13**, **19**, **20**, **25**, **29**, and **32**) measured by the MTT assay with the presence of LPS (A) and without LPS (B). Data are presented as means ± standard deviation from samples with three replicated experiments (*n* = 3). Statistical analysis was performed with a one-way analysis of variance with multiple comparisons and the significance was noted as *****p* < 0.0001 vs. the control group.

**
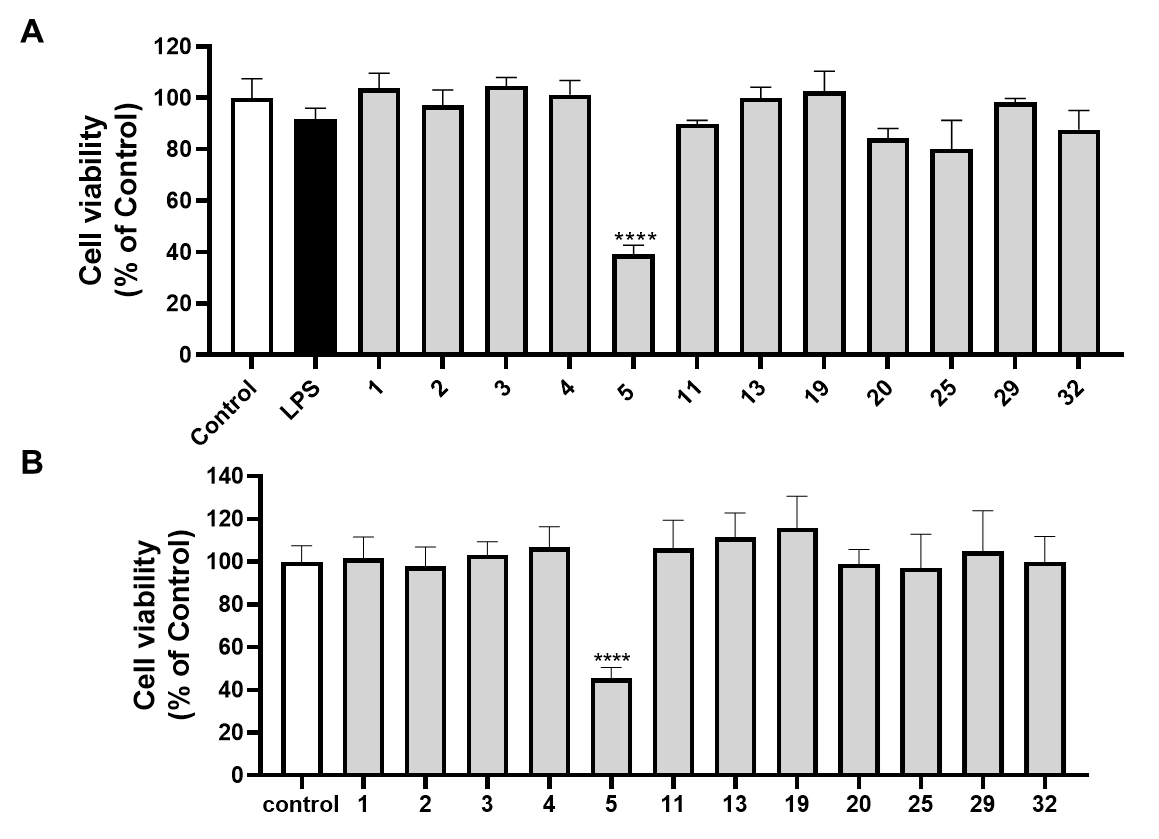
**

**Figure S2.** Anti-inflammatory effects of phytochemicals isolated from hemp roots. Inflammasome activation in THP-cells was induced by the stimulation with LPS-nigericin. Effects of test compounds (at 50 µM) on the production of pro-inflammatory cytokines including IL-1β. Data are presented as means ± standard deviation from samples with three replicated experiments (*n* = 3). Statistical analysis was performed with a one-way analysis of variance with multiple comparisons and the significance was noted as: **p* < 0.005, *****p* < 0.0001 vs. the model (LPS-nigericin stimulated) group and ####*p* < 0.0001 vs. the control group.
